# Dynamic Monitoring of *Chilo suppressalis* Resistance to Insecticides and the Potential Influencing Factors

**DOI:** 10.3390/plants14050724

**Published:** 2025-02-27

**Authors:** Wujia Mo, Qiang Li, Zhongxian Lu, Farman Ullah, Jiawen Guo, Hongxing Xu, Yanhui Lu

**Affiliations:** 1 State Key Laboratory for Managing Biotic and Chemical Threats to the Quality and Safety of Agro-Products, Institute of Plant Protection and Microbiology, Zhejiang Academy of Agricultural Sciences, Hangzhou 310021, China; 15682539209@163.com (W.M.); liqiang1120@zju.edu.cn (Q.L.); farmanullah787@gmail.com (F.U.); guojiawen91@126.com (J.G.); 2 State Key Laboratory of Rice Biology and Ministry of Agricultural and Rural Affairs, Key Laboratory of Molecular Biology of Crop Pathogens and Insect Pests, Institute of Insect Sciences, Zhejiang University, Hangzhou 310058, China; luzxmh@163.com

**Keywords:** *Chilo suppressalis*, insecticide resistance, detoxifying enzyme, climate

## Abstract

*Chilo suppressalis* is one of the most important rice pests worldwide, and chlorantraniliprole, abamectin, and methoxyfenozide have been widely used to control this pest in China. However, the control efficiency in the field has dramatically decreased in recent years. Therefore, assessing the impacts of different factors on *C. suppressalis* resistance is essential for maintaining control effectiveness and managing resistant populations. Herein, we investigated insecticide resistance and its potential influencing factors (biotic and abiotic factors) in *C. suppressalis* field populations, using bioassays and biochemical and molecular diagnostic approaches. The results showed that the resistance levels of most field populations of *C. suppressalis* have evolved to moderate-to-high levels to the tested insecticides. The toxicity correlation analysis indicated that there was a significant positive correlation between the resistance levels of abamectin and methoxyfenozide, whereas GST activity was positively correlated with abamectin and methoxyfenozide resistance in *C. suppressalis*. EST and P450 activities showed significantly positive correlation with the resistance of chlorantraniliprole and methoxyfenozide, while the increase in temperature enhanced EST enzyme activity and was positively correlated with the evolution of resistance to methoxyfenozide. Overall, our study provides a systematic understanding of the dynamic resistance status and its influencing factors of *C. suppressalis* to insecticides. These findings will help clarify the resistance levels and the influencing factors in the resistance development of *C. suppressalis*, providing a theoretical basis for the resistance management of this insect species.

## 1. Introduction

The rice stem borer, *Chilo suppressalis* (Walker) (Lepidoptera: Crambidae), is one of the most devastating and perennial rice pests, mainly distributed in the Yangtze River Basin and the main rice areas in the south of China, especially in the costal and plain areas along the Yangtze River [1,2]. Zhejiang Province stands out as a typical example, one of the most important rice-producing regions in China. The larvae feed on stems during all rice growth stages, causing “deadhearts” at the vegetative stage and “whiteheads” at the reproductive stage [3,4,5], which pose a significant threat to rice production and food security [6,7,8,9]. In recent years, shifts in rice cultivation practices, the widespread adoption of hybrid varieties, and changes in climate conditions have collectively contributed to a surge in the frequency of *C. suppressalis* outbreaks [4,7,10]. The control of *C. suppressalis* has failed in some areas of Zhejiang Province, which poses a serious threat to sustainable rice production.

Meanwhile, insecticide resistance is influenced by multiple factors, including biotic factors (enzyme activity and gene mutations) and abiotic factors (external climate and changes cross-resistance) [11,12,13]. For the biotic factors, insects may increase enzyme activities, which can render pesticides ineffective, enabling insects to survive [14]. Over time, resistance-associated genetic mutations can accumulate in the populations, leading to widespread resistance. For the abiotic, global climate change significantly affects many organisms including insects, via rising temperatures, changes in ultraviolet radiation levels, and unpredictable changes in precipitation [12,15]. Environmental alterations caused by climate may lead to changes in the efficacy of insecticides, as studies have shown that temperature can affect the toxicity of many insecticides used for pest management, resulting in reduced efficacy [16]. Furthermore, the evolutionary response to climate change may lead to rapid changes in the allele frequencies of genes related to insecticide resistance, which is due to the pleiotropic effects of these insecticide resistance genes on other traits, such as heat resistance or desiccation tolerance [17]. Therefore, insecticide resistance and climate adaptation will co-evolve. In addition, cross-resistance is also an important factor to be noticed in monitoring insecticide resistance. The occurrence of cross-resistance is related to the species of pests, the genetic background of the field pest populations, and the history of field medication [11,18]. Various insecticides with different modes of action are most used to delay resistance development in pest management [19]. Therefore, it is crucial to understand the cross-resistance mechanisms for managing key insect pests [20].

At present, due to the destructiveness and rapid expansion of *C. suppressalis*, agricultural, physical, and biological control methods are insufficient to meet production needs; so, its control relies heavily on pesticides. Hence, the control of *C. suppressalis* primarily depends on the application of insecticides in the long term, and insecticide resistance is an important factor contributing to outbreaks [21,22,23]. Previous studies reported that field-evolved resistance of *C. suppressalis* to some conventional insecticides such as organophosphates, benzoylphenyl urea, diamides, and abamectin has already occurred in China [4,5,7,24,25,26,27,28,29]. Some field populations of *C. suppressalis* have developed extremely high levels of resistance to monosultap and triazophos and moderate or high levels of resistance to chlorpyrifos and chlorantraniliprole [4,7,30,31,32]. In recent years, the anthranilic diamide insecticide chlorantraniliprole, macrocyclic lactone insecticide abamectin and the bisacylhydrazine methoxyfenozide have been involved in the control of stem borer. Chlorantraniliprole selectively targets insect ryanodine receptors (RyRs) that causes uncontrolled Ca^2+^ release from the endoplasmic reticulum to the cytoplasm, leading to the cessation of feeding, paralysis, and finally death of an insect [33,34,35]. After removing insecticides with ecological risk and strong resistance selection pressure, the application of abamectin has become one of the main control methods by influencing the membrane chloride conductance on a variety of ligand-gated chloride channels [36,37]. Methoxyfenozide binds to the ecdysone receptor protein and leads to ecdysis, starvation, dehydration, and ultimately death with difficulty in developing resistance and high selection [38,39].

A comprehensive understanding of the factors influencing resistance development is essential for Integrated Pest Management (IPM) and Insect Resistance Management (IRM) [40,41]. Enhanced detoxification of metabolic enzymes and the insensitivity of target sites are key mechanisms of insect resistance to insecticides [7,42,43]. Increased metabolic resistance to insecticides is associated with the enhanced activities of detoxifying enzymes, including esterase (ESTs), glutathione S-transferases (GSTs), and cytochrome P450 monooxygenase (P450s) [43,44]. In addition, ESTs and GSTs are usually associated with resistance to organophosphate insecticides, and the enhancement of P450s activity is linked with diamide resistance [45,46,47,48]. The primary resistance mechanism of *C. suppressalis* against chlorantraniliprole includes point mutations of the ryanodine receptor and metabolic detoxification [4,49].

Monitoring insecticide resistance should be an essential component of IPM. To provide basic data for the resistance management strategies, it is crucial to evaluate the resistance levels of *C. suppressalis* against commonly used insecticides in rice cultivation. In this study, we conducted resistance responses to chlorantraniliprole, abamectin, and methoxyfenozide in different geographical populations collected from paddy fields and analyzed biotic and abiotic influence factors to resistance development, which may provide a theoretical basis for the resistance management of *C. suppressalis*.

## 2. Results

### 2.1. Toxicological Responses to Abamectin, Chlorantraniliprole, and Methoxyfenozide

**Figure 1:**
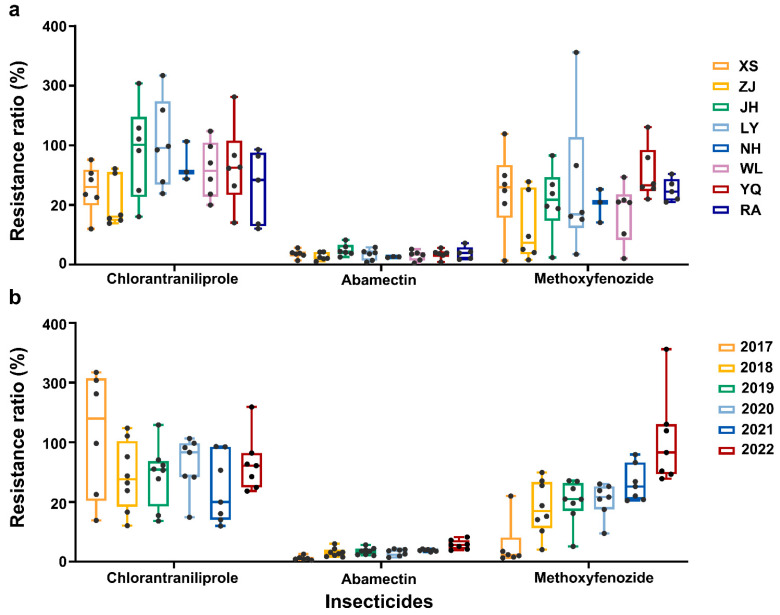
The range of resistance levels in the different populations of *C. suppressalis* to chlorantraniliprole, abamectin, and methoxyfenozide. (**a**) The range of resistance levels in different field populations collected from 2017 to 2022; (**b**) the range of resistance levels in different field populations collected from the same year.

The toxicity bioassay showed that the LY-2017, WL-2017, and YQ-2017 populations were susceptible to abamectin (resistance ratio (RR) < 5.0), with LC_50_ values ranging from 2.38 mg/L (WL-2017) to 4.03 mg/L (LY-2017). Six populations (ZJ-2017, XS-2017, LY-2020, WL-2018, RA-2018, and ZJ-2020) exhibited low-level resistance (5.0 ≤ RR < 10.0) to abamectin. Another thirty-five populations showed moderate resistance (10.0 ≤ RR < 100.0) to abamectin with LC_50_ values ranging from 9.79 mg/L (RA-2019) to 38.47 mg/L (JH-2022), leading to the RRs ranging between 10.52 and 41.32 (Figure 1 and Table S1).

The toxicities of chlorantraniliprole towards various regions populations collected from Zhejiang Province from 2017 to 2022 are listed in Table S2. The results showed that the resistance levels of *C. suppressalis* to chlorantraniliprole ranged widely. Based on the susceptibility baseline, all the field-collected populations showed moderate-to-high levels of chlorantraniliprole resistance with RRs of 59.96- to 317.12-fold (Figure 1). The results of the toxicological responses showed that ten populations, including XS-2021, ZJ-2017~ZJ2020, JH-2021, WL-2021, YQ-2021, RA-2018, and RA-2019 had moderate resistance (10.0 ≤ RR < 100.0) to chlorantraniliprole, with LC_50_ values varying from 83.52 mg/L (XS-2021) to 139.21 mg/L (WL-2021), leading to RRs ranging from 59.96 to 99.94. Another thirty-four populations were highly resistant (RR > 100) to chlorantraniliprole, with LC_50_ values varying from 156.93 mg/L (XS-2017) to 441.75 mg/L (LY-2017), leading to RRs ranging from 112.66- to 317.12-fold, compared to the susceptible baseline.

The results of the toxicological responses showed that the XS-2017 and ZJ-2017 populations had low-level resistance to methoxyfenozide. Thirteen populations showed moderate resistance (10.0 ≤ RR < 100.0) to methoxyfenozide, with LC_50_ values varying from 7.54 mg/L (WL-2017) to 71.49 mg/L (JH-2019), leading to RRs ranging from 10.34 to 98.07, compared to the susceptible baseline. Another twenty-nine populations were highly resistant (RR > 100) to methoxyfenozide, with LC_50_ values varying from 74.84 mg/L (XS-2021) to 259.38 mg/L (LY-2022), leading to RRs ranging between 102.63 and 355.80 (Figure 1 and Table S3).

### 2.2. EST, GST, and Cytochrome P450 Activities

**Figure 2:**
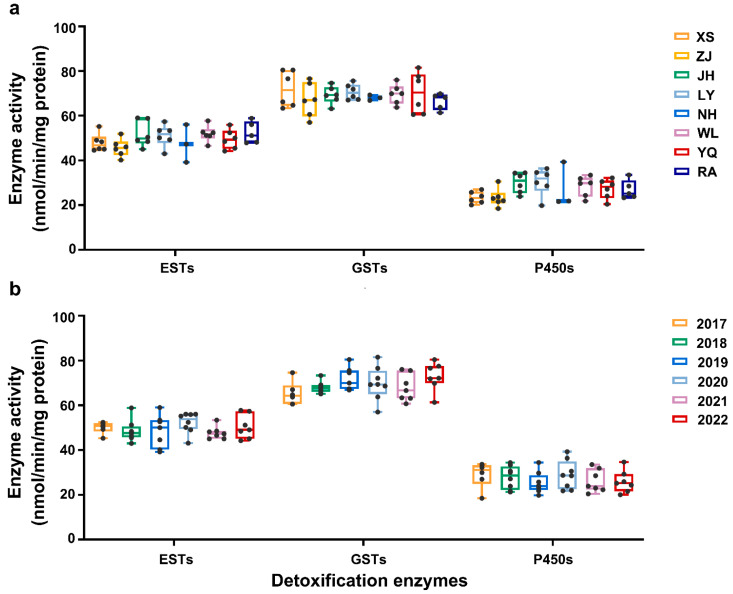
The range of enzyme activities (ESTs, GSTs, and P450s) in the different populations of *C. suppressalis*. (**a**) The range of resistance levels in different field populations collected from 2017 to 2022; (**b**) the range of resistance levels in different field populations collected from the same year.

The results showed that the EST activities ranged from 39.20 (NH population in 2019) to 59.02 (JH population in 2019) nmol/min/mg protein. A marked difference was observed in 2020, where the YQ and RA populations exhibited significantly higher activities than the ZJ populations. However, there were no significant differences in the field populations of *C. suppressalis* among different regions in the same year (Figure 2 and Table S4).

As shown in Figure 2 and Table S5, there was no significant difference in GST activity among different regions and years. From 2017 to 2022, the lowest activity of the tested populations was 49.12 nmol/min/mg protein, observed in the ZJ population in 2020, while the highest was 94.62 nmol/min/mg protein appearing also in the ZJ population in 2022.

P450 activity showed significant variation across different regions and years. The P450 activities ranged from 15.23 (XS population in 2018) to 45.33 (NH in 2020) nmol/min/mg protein. In the ZJ population, the P450 activity in 2018 was significantly higher than in other years. In addition, the activity of P450 of the LY population was substantially lower in 2017 and 2022 compared with the activity in 2019. In 2020, the NH population showed significantly higher P450 activity than other populations, while the WL population had the lowest activity this year. In 2021 and 2022, the YQ and XS populations displayed the lowest P450 activity, whereas the LY and RA populations exhibited the highest. (Figure 2 and Table S6).

### 2.3. Correlations of Resistance to Three Insecticides

The resistance of *C. suppressalis* against three insecticides was examined. The results showed that the populations had developed different levels of resistance to each tested insecticide (Table 1). Among the three insecticides, chlorantraniliprole showed a relatively low level of correlation for the tested populations with abamectin and methoxyfenozide. The pair-wise correlation coefficient comparison indicated that the logLC_50_ values significantly correlated between abamectin and methoxyfenozide (*R* = 0.698, *p* < 0.001).

### 2.4. Correlations Between Detoxification Enzyme Activities and Insecticide Toxicities

**Figure 3:**
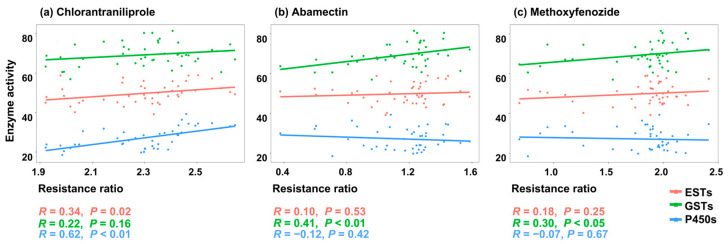
Pair-wise correlation coefficient between LogLC_50_ values of the tested insecticides in the *C. suppressalis* field populations and enzyme activities.

To determine the role of detoxification enzymes in the insecticide resistance of *C. suppressalis*, the correlation coefficient between the LC_50_ values of *C. suppressalis* to insecticides and the enzyme activities of ESTs, GSTs, and P450s were analyzed, respectively. The detoxification enzyme activities were detected in forty-four populations of *C. suppressalis*. The results showed a significant positive correlation between the EST/P450 activities and the LC_50_ values of *C. suppressalis* to chlorantraniliprole (*R* = 0.340, *p* = 0.024/*R* = 0.621, *p* < 0.001). The GST activity was positively related to the resistance levels of *C. suppressalis* against abamectin (*R* = 0.410, *p* = 0.006) and methoxyfenozide (*R* = 0.302, *p* = 0.046), while no significant correlation was observed with chlorantraniliprole). However, there were no significant correlations between P450 activities and the resistance levels of abamectin and methoxyfenozide (Figure 3 and Table 2).

### 2.5. Correlations Between the Resistance Ratio and Climate Factors

**Figure 4:**
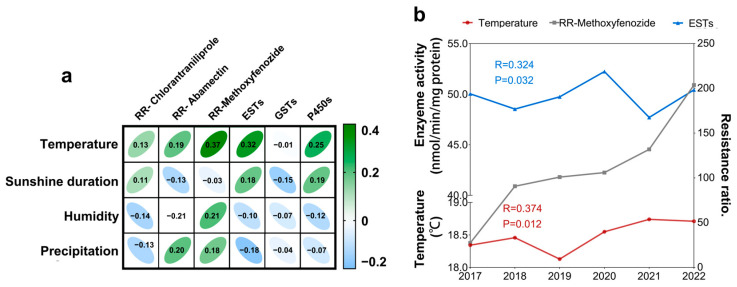
Pair-wise correlation coefficient between resistance ratio of tested insecticides in the *C. suppressalis* field populations and climate factors (**a**) Thermal map illustrating the correlation between climate factors (temperature, sunshine duration, humidity, and precipitation) and insecticide resistance, as well as enzyme activities; (**b**) line graph of temperature, ESTs, and resistance ratio of methoxyfenozide.

As shown in Figure 4, the surface temperature was significantly positively correlated with the enzyme activity of ESTs (*R* = 0.324, *p* = 0.032) and the resistance ratio of methoxyfenozide (*R* = 0.374, *p* = 0.012), respectively. No significant correlations were found between other factors (sunshine duration, humidity and precipitation) and resistance and the enzyme activity of *C. suppressalis* population.

## 3. Discussion

This study elucidated the role of detoxification enzymes (ESTs, GSTs, and P450s) in *C. suppressalis* resistance to these insecticides. Chlorantraniliprole, abamectin, and methoxyfenozide are widely used to control this key pest [4]. Additionally, we analyzed the correlation between the detoxification enzyme activity, cross-resistance, and external climate change and the resistance of *C. suppressalis* to three insecticides. These findings provide valuable insights to better understand the resistance development and their potential influencing factors, which will ultimately help in the resistance management of *C. suppressalis*.

The results showed that the *C. suppressalis* collected from forty-four field populations exhibited different resistance levels to chlorantraniliprole, abamectin, and methoxyfenozide, which reflect differences in the insecticide exposure history and selection pressure across regions. All the tested field populations of *C. suppressalis* exhibited moderate-to-high levels of resistance to chlorantraniliprole, with no significant changes [4,29]. This may be due to the decreasing amount of pesticides under the national policies, which has suppressed the evolution of resistance in *C. suppressalis*. Previous studies showed that Zhejiang populations of *C. suppressalis* developed low levels of resistance to abamectin [2,50]. However, our findings indicate that most of the tested populations have developed moderate levels of resistance to abamectin, likely due to the increasing use of insecticides [2,34]. Similarly, our results revealed that most of the field populations of *C. suppressalis* developed moderate or high resistance to methoxyfenozide, consistent with previous reports of resistance in *Musca domestica* and *Helicoverpa armigera* against these insecticides [51]. In Zhejiang, abamectin and methoxyfenozide have been widely used as alternative control agents, primarily due to the increasing resistance developed against chlorantraniliprole, which could explain the subsequent rise in resistance levels observed for these two insecticides. Interestingly, significant correlations were observed in LogLC_50_ between abamectin and methoxyfenozide, which was consistent with previous studies, and this phenomenon might be due to a similar mechanism of action [7,28]. Therefore, it is crucial to avoid the evolution of cross-resistance among insecticides which might lead to multi-resistance. The variation in resistance levels in different populations may be related to several reasons, such as the history of insecticide application, the climate in different geographical regions, detoxification enzymes, and insecticide cross-resistance [4,52]. It was suggested that insecticides with no cross-resistance should be substituted to delay the evolution of resistance development [27].

ESTs, GSTs, and P450s are the three major enzyme families involved in the catabolism and/or sequestration of pesticides [53,54], playing an important role in the development of insecticide resistance. Previous studies have reported that the increased activities of ESTs and P450s could contribute to the resistance of chlorantraniliprole and abamectin in *C. suppressalis* [4,7,29,34,55,56]. Our experimental findings aligned seamlessly with these observations, reinforcing the correlation that such enzyme activities (ESTs and P450s) were indeed associated with insecticide resistance. Furthermore, the GSTs were significantly correlated with the resistance of *C. suppressalis* to abamectin and/or methoxyfenozide, which indicated that GSTs might be a potential action mechanism in the metabolism of these two insecticides. Based on the correlation analysis between enzyme activities and insecticides toxicities, the resistant insect populations would show cross-resistance to other compounds metabolized by GSTs [57,58]. Moreover, a significant correlation was analyzed between the enzyme activities and resistance levels of insecticides in the field populations of *C. suppressalis*. Correspondingly, tebufenozide-resistant *Plutella xylotslla* showed obvious cross-resistance to abamectin, which may be due to the occurrence of enhanced metabolism [59]. Therefore, it is crucial to avoid the evolution of cross-resistance among insecticides, which might lead to multi-resistance.

Some studies suggested that climate change accelerates the evolution of pesticide resistance by altering environmental factors such as temperature and humidity [17]. ESTs can mediate the evolution of resistance to chlorantraniliprole and methoxyfenozide. Furthermore, ESTs were significantly positively correlated with the increase in temperature, suggesting that climate warming could accelerate the evolution of resistance to methoxyfenozide in striped stem borer larvae by enhancing EST activity.

## 4. Materials and Methods

### 4.1. C. suppressalis Populations

**Figure 5:**
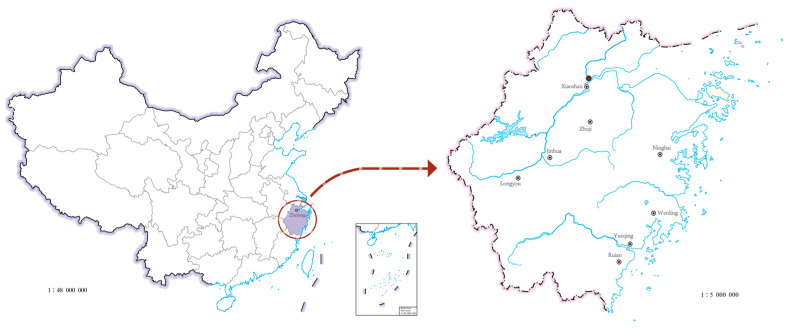
Sampling sites of *C. suppressalis* from Zhejiang Province in China. The regions include Xiaoshan (the population code is XS), Zhuji (ZJ), Longyou (LY), Jinhua (JH), Ninghai (NH), Wenling (WL), Yueqing (YQ), and Ruian (RA).

A total of forty-four populations of *C. suppressalis* (egg masses, larvae, or adults) were collected from rice fields in Zhejiang provinces, China, from 2017 to 2022 (Figure 5 and Table S7). The collected insects were mass-reared in an artificial atmospheric phenomena simulator under standard conditions of 27 ± 1 °C and 70–80% relative humidity with a photoperiod of 16 h:8 h (L:D). The larvae were reared on an artificial diet, and the adults were fed with a 10% honey solution. The third-instar larvae of the F1 generation were used for the bioassay.

### 4.2. Chemicals

The insecticides used in the larvae bioassays were technical grade compounds. Chlorantraniliprole (95.0%, 3-bromo-4′-chloro-1-(3-chloro-2-pyridyl)-2′-methyl-6′-(methylcarbamoyl)pyrazole-5-carboxanilide), abamectin (98.0%), and methoxyfenozide (98.2%, N-tert-butyl-N′-(3-methoxy-o-toluoyl)-3,5-xylohydrazide) were provided by Prof. Xiwu Gao of China Agricultural University. The stock solution of chlorantraniliprole was dissolved in dimethyl sulfoxide (DMSO), while the abamectin and methoxyfenozide stock solutions were dissolved in acetone. Both analytical grade DMSO and acetone were obtained from Sinopharm Chemical Reagent Co., Ltd. (Shanghai, China). Total enzyme activity kits were obtained from Nanjing Jiancheng Bioengineering Institute (Nanjing, China).

### 4.3. Bioassays

The insecticide toxicity to third-instar larvae was evaluated with a seedling-dip bioassay [4]. Briefly, seedlings of 25 cm height (ca. 3 weeks) were used to conduct the bioassays. The insecticides were adjusted to the required concentrations (serially diluted two-fold to five) with distilled water containing 0.1% (*v*/*v*) Triton X-100 (Beijing Solarbio Science and Technology Co. Ltd., Beijing, China). The seedlings were cut into 6 cm sections, dipped in each solution for 10 s, and then air dried. Fifteen stem sections were placed into a bottle (200 mL) containing 4 filter paper disks moistened with 3 mL distilled water and 20 third-instar larvae. Each concentration was performed with three replicates, for a total of 60 larvae. The mortality was recorded 6 days after chlorantraniliprole treatment and 3 days after treatment with abamectin and methoxyfenozide. Larvae were considered dead if they did not move at all when gently probed with a brush. The control stems were treated with 0.1% Triton X-100 solution, and the mortality of the controls was <10%.

### 4.4. Detoxification Enzyme Activity Assays

Forty-four field populations of *C. suppressalis* were sampled to assess the insecticide resistance at the biochemical level. Five third-instar larvae from each population were homogenized in 2.0 mL ice-cold homogenization buffer and centrifuged at 4 °C, 10,000× *g* (Eppendorf centrifuge 5417R, Hamburg, Germany) for 15 min. The supernatant fluid served as the enzyme source for measuring the activity of enzymes. The protein concentrations were analyzed using BSA (Sigma-Aldrich, St Louis, MO, USA) as the standard [60]. All samples were measured with a SpectraMax 190 microplate reader (Molecular Devices, Sunnyvale, CA, USA) with three technical replicates. The specific activity of ESTs, GSTs, and P450s-dependent monooxygenases (O-demethylases) were calculated in nmol/min/mg protein.

### 4.5. Climatic Data Sources

Climate data from https://neo.gsfc.nasa.gov/ included the near-surface temperature, 10 m wind speed, relative humidity, and precipitation from 2017 to 2022.

### 4.6. Data Analysis

The LC_50_ values were calculated using the Polo-Plus program (v2.0) [61]. The resistance ratio was calculated as the LC_50_ value of a field population divided by the pre-determined baseline LC_50_ value of a susceptible strain. The insecticide resistance levels were classified as susceptible (RR < 5.0), low resistance (5.0 ≤ RR < 10.0); moderate resistance (10.0 ≤ RR < 100.0); high resistance (RR ≥ 100.0) [27]. Statistical significance was determined by one-way ANOVA and Fisher’s exact probability test using the SPSS Statistics 20 software package (IBM Corp, 2020). The reported *p* values were adjusted to account for multiple comparisons with a Bonferroni correction. *p* < 0.05 or *p* < 0.01 was considered to be statistically significant. Correlation analysis was also performed by SPSS Statistics software package with Spearman correlation analysis, and graphs were produced with Graphpad Prism 9.0 and Origin 2022.

## 5. Conclusions

Collectively, our study offered a comprehensive insight into the dynamic resistance levels exhibited by *C. suppressalis*, along with an exploration of the potential influencing factors (detoxification cross-resistance, enzyme activity, and climate change). The correlation analysis revealed a significant correlation between the resistance ratios of abamectin and methoxyfenozide, hinting at the likelihood of cross-resistance. This cross-resistance may stem from the common involvement of GSTs enzymes in their respective detoxification mechanisms. The activity of EST enzymes displayed a notable positive correlation with the resistance to both chlorantraniliprole and methoxyfenozide. Furthermore, an increase in temperature served as an inducer, enhancing the activity of these ESTs enzymes. Therefore, alternative management strategies such as mating disruption and biological control should be combined with chemical insecticides to decrease resistance and achieve sustainable control of *C. suppressalis*.

## Figures and Tables

**Table 1:** Pair-wise correlation coefficient comparison between LogLC_50_ values of the tested insecticides in the *C. suppressalis* field populations.

		Chlorantraniliprole	Abamectin
Abamectin	*R* value	−0.017	
	*p* value	0.914	
Methoxyfenozide	*R* value	0.055	0.698 **
	*p* value	0.724	<0.001

** Positive correlation between LC_50_ values of insecticides and enzyme activity at the 99% significance level.

**Table 2:** Pair-wise correlation coefficient between LogLC_50_ values of the tested insecticides in the *C. suppressalis* field populations and enzyme activities.

		Chlorantraniliprole	Abamectin	Methoxyfenozide
ETSs	*R* value	0.340 *	0.097	0.177
	*p* value	0.024	0.531	0.249
GSTs	*R* value	0.216	0.410 **	0.302 *
	*p* value	0.160	0.006	0.046
P450s	*R* value	0.621 **	−0.125	−0.066
	*p* value	<0.001	0.420	0.669

** Positive correlation between LC_50_ values of insecticides and enzyme activity at the 99% significance level. * Positive correlation between LC_50_ values of insecticides and enzyme activity at the 95% significance level.

## Data Availability

Data are contained within the article.

## Supplementary Materials

The following supporting information can be downloaded at https://www.mdpi.com/article/10.3390/plants14050724/s1, Table S1: Resistance levels of field populations of *Chilo suppressalis* to chlorantraniliprole by seedling-dip bioassays.; Table S2: Resistance levels of field populations of *C. suppressalis* to abamectin by seedling-dip bioassays.; Table S3: Resistance levels of field populations of *C. suppressalis* to methoxyfenozide by seedling-dip bioassays.; Table S4: ESTs enzyme activities of *C. suppressalis* in eight field populations.; Table S5: GSTs enzyme activities of *C. suppressalis* in eight field populations.; Table S6: P450s enzyme activities of *C. suppressalis* in eight field populations; Table S7: Sampling sites, collection dates, and developmental stages of *Chilo suppressalis* collected from fields.
