# Supplementary material for: Dynamic Monitoring of Chilo suppressalis Resistance to Insecticides and the Potential Influencing Factors
Source: Plants (Basel). 2025 Feb 27;14(5):724. doi: 10.3390/plants14050724 (PMC11901930; doi:10.3390/plants14050724)
Supplement: Supplementary file 1 [file plants-14-00724-s001.zip › plants-3403840-supplementary.pdf]

Table S1 Resistance levels of field populations of *Chilo suppressalis* to abamectin by seedling dip bioassays.

Population	Slope ± SE	χ^2 (df) a	LC_{50} (95 % FL) (mg/L)	RR b
XS-2017	2.17±0.22	0.12 (3)	6.45 (5.42-7.77)	6.93
XS-2018	1.96±0.22	1.86 (3)	14.90 (12.14-18.00)	16.00
XS-2019	2.86±0.27	1.47 (3)	26.39 (22.82-30.70)	28.35
XS-2020	2.43±0.24	1.91 (3)	18.71 (15.88-22.02)	20.10
XS-2021	2.14±0.31	2.03 (3)	16.67 (12.75-21.46)	17.91
XS-2022	2.50±0.24	1.80 (3)	17.76 (15.12-20.83)	19.08
ZJ-2017	2.34±0.23	0.38 (3)	5.11 (4.32-6.04)	5.49
ZJ-2018	2.28±0.23	5.64 (3)	9.92 (6.50-14.89)	10.66
ZJ-2019	1.92±0.21	0.33 (3)	11.41 (9.41-13.94)	12.26
ZJ-2020	2.54±0.25	2.84 (3)	9.12 (7.78-10.68)	9.80
ZJ-2021	2.11±0.22	0.56 (3)	20.05 (16.74-24.01)	21.54
ZJ-2022	2.33±0.23	0.92 (3)	19.64 (16.59-23.21)	21.10
JH-2017	2.44±0.34	4.51 (3)	11.88 (8.52-17.06)	12.76
JH-2018	2.24±0.19	4.74 (3)	28.02 (21.01-39.05)	30.10
JH-2019	2.20±0.22	0.58 (3)	20.76 (17.43-24.77)	22.30
JH-2020	2.42±0.24	1.66 (3)	19.12 (16.22-22.49)	20.54
JH-2021	2.50±0.34	0.35 (3)	17.60 (13.93-22.11)	18.90
JH-2022	1.94±0.22	1.41 (3)	38.47 (31.48-49.00)	41.32
LY-2017	2.05±0.22	1.15 (3)	4.03 (3.33-4.84)	4.33
LY-2018	2.00±0.21	0.09 (3)	20.25 (16.78-24.46)	21.75
LY-2019	2.32±0.23	3.72 (3)	16.80 (12.03-22.94)	18.05
LY-2020	1.93±0.31	1.34 (3)	6.82 (4.97-8.95)	7.33
LY-2021	2.28±0.28	0.32 (3)	18.58 (15.63-22.02)	19.96
LY-2022	1.97±0.21	1.40 (3)	27.10 (22.44-33.28)	29.11
NH-2018	2.15±0.23	2.50 (3)	12.75 (10.47-15.24)	13.69
NH-2019	2.68±0.26	1.13 (3)	11.18 (9.61-13.04)	12.01
NH-2020	2.70±0.26	1.54 (3)	12.07 (10.38-14.10)	12.96
WL-2017	2.00±0.24	3.23 (3)	2.38 (1.49-3.30)	2.56
WL-2018	2.49±0.24	3.65 (3)	7.36 (5.31-9.83)	7.91
WL-2019	2.18±0.23	0.59 (3)	16.46 (13.72-19.58)	17.68
WL-2020	2.70±0.26	1.67 (3)	18.20 (15.62-21.16)	19.55

WL-2021	1.97±0.31	1.29 (3)	15.34 (11.39-20.05)	16.48
WL-2022	2.16±0.22	0.47 (3)	24.20 (20.31-29.08)	25.99
YQ-2017	2.11±0.22	1.64 (3)	3.95 (3.27-4.72)	4.24
YQ-2018	2.46±0.25	1.64 (3)	14.66 (12.38-17.21)	15.75
YQ-2019	2.48±0.24	3.96 (3)	18.42 (13.41-25.19)	19.79
YQ-2020	2.48±0.37	1.07 (3)	16.97 (13.50-22.08)	18.23
YQ-2021	2.23±0.32	0.28 (3)	18.98 (14.75-24.35)	20.39
YQ-2022	1.76±0.20	0.29 (3)	26.10 (21.25-32.67)	28.03
RA-2018	2.21±0.23	1.03 (3)	8.09 (6.75-9.61)	8.69
RA-2019	2.85±0.27	1.98 (3)	9.79 (8.46-11.34)	10.52
RA-2020	2.49±0.24	6.14 (3)	20.18 (13.53-30.55)	21.68
RA-2021	2.54±0.34	0.35 (3)	18.21 (14.45-22.81)	19.56
RA-2022	2.41±0.25	1.50 (3)	33.58 (28.46-40.40)	36.07

a Chi-square testing linearity of dose-mortality responses.

b Resistance ratio. The susceptibility baseline to abamectin (0.931 mg/L) was from reference of Lu et al. (2017).

Table S2 Resistance levels of field populations of *C. suppressalis* to chlorantraniliprole by seedling dip bioassays.

Population	Slope ± SE	χ^2 (df) a	LC_{50} (95 % FL) (mg/L)	RR b
XS-2017	1.87±0.21	5.93 (3)	156.93 (88.09-252.22)	112.66
XS-2018	2.41±0.24	1.87 (3)	245.26 (208.46-290.10)	176.06
XS-2019	2.40±0.24	2.11 (3)	213.47 (164.69-268.82)	153.24
XS-2020	2.39±0.24	3.33 (3)	163.93 (120.70-218.24)	117.68
XS-2021	2.25±0.32	0.41 (3)	83.520 (64.56-106.66)	59.96
XS-2022	2.41±0.21	7.94 (3)	198.43 (122.52-328.76)	142.45
ZJ-2017	2.21±0.23	2.02 (3)	96.68 (81.15-115.16)	69.40
ZJ-2018	2.07±0.22	2.20 (3)	115.89 (96.64-139.83)	83.19
ZJ-2019	1.95±0.21	1.03 (3)	108.04 (89.27-131.35)	77.56
ZJ-2020	2.45±0.24	0.83 (3)	103.83（88.36-122.14）	74.54
ZJ-2021	1.68±0.21	1.04 (3)	213.54 (169.39-287.87)	153.30
ZJ-2022	2.07±0.22	0.31 (3)	224.15 (186.87-270.43)	160.91
JH-2017	2.89±0.27	2.34 (3)	423.31 (366.24-489.85)	303.88
JH-2018	1.43±0.16	4.90 (3)	293.44 (192.52-520.08)	210.65
JH-2019	1.99±0.22	0.60 (3)	319.12 (264.12-395.62)	229.09

JH-2020	2.38±0.24	0.88 (3)	266.33 (226.09-316.65)	191.19
JH-2021	2.20±0.32	2.68 (3)	112.13 (87.23-144.93)	80.50
JH-2022	2.02±0.23	0.42 (3)	173.55 (143.57-216.16)	124.59
LY-2017	2.16±0.25	1.90 (3)	441.75 (365.23-557.67)	317.12
LY-2018	1.90±0.21	3.80 (3)	166.06 (111.04-240.05)	119.21
LY-2019	2.53±0.24	0.47 (3)	193.47 (164.98-226.58)	138.89
LY-2020	2.35±0.34	0.14 (3)	276.76 (218.72-358.46)	198.68
LY-2021	2.19±0.23	0.81 (3)	268.17 (225.42-322.91)	192.51
LY-2022	2.00±0.22	0.24 (3)	361.02 (297.85-452.48)	259.17
NH-2018	2.48±0.24	5.89 (3)	200.40 (135.04-300.04)	143.86
NH-2019	2.05±0.21	1.26 (3)	216.23 (179.92-260.82)	155.23
NH-2020	2.29±0.24	1.32 (3)	287.56 (242.86-345.33)	206.43
WL-2017	1.51±0.21	1.93 (3)	276.17 (209.2-411.23)	198.26
WL-2018	2.84±0.28	3.88 (3)	311.70 (236.53-427.25)	223.76
WL-2019	2.28±0.23	2.40 (3)	237.81 (200.88-283.44)	170.72
WL-2020	2.12±0.22	3.65 (3)	199.71 (141.72-279.60)	143.37
WL-2021	2.65±0.37	1.98 (3)	139.21 (109.86-173.01)	99.94
WL-2022	2.17±0.22	0.02 (3)	164.91 (137.40-196.42)	118.38
YQ-2017	1.91±0.22	1.92 (3)	391.51 (319.36-501.56)	281.06
YQ-2018	2.56±0.25	4.67 (3)	184.09 (129.90-256.18)	132.15
YQ-2019	2.56±0.25	2.95 (3)	225.02 (192.53-263.90)	161.54
YQ-2020	1.96±0.30	0.24 (3)	255.11 (194.78-343.14)	183.14
YQ-2021	2.87±0.38	0.93 (3)	98.11 (79.44-120.94)	70.43
YQ-2022	1.98±0.23	1.24 (3)	227.65 (185.26-295.09)	163.42
RA-2018	1.98±0.21	5.07 (3)	84.15 (52.26-127.58)	60.41
RA-2019	2.49±0.24	0.52 (3)	95.17 (81.01-111.67)	68.32
RA-2020	1.86±0.30	3.11 (3)	197.80 (120.75-358.98)	142.00
RA-2021	2.34±0.34	1.24 (3)	268.79 (212.36-348.05)	192.96
RA-2022	2.08±0.22	3.02 (3)	253.81 (188.26-354.73)	182.20

a Chi-square testing linearity of dose-mortality responses.

b Resistance ratio. The susceptibility baseline to chlorantraniliprole (1.393 mg/L) was from reference of Gao et al. (2013).

Table S3 Resistance levels of field populations of *C. suppressalis* to methoxyfenozide by seedling dip bioassays.

Population	Slope ± SE	χ^2 (df) a	LC_{50} (95 % FL) (mg/L)	RR b
XS-2017	2.25±0.23	0.12 (3)	4.88 (4.11-5.80)	6.69
XS-2018	1.74±0.20	2.05 (3)	109.06 (88.46-135.21)	149.60
XS-2019	2.04±0.22	1.03 (3)	98.24 (81.58-118.29)	134.76
XS-2020	2.19±0.22	3.29 (3)	90.61 (66.08-123.28)	124.29
XS-2021	2.29±0.33	2.88 (3)	74.82 (57.76-95.15)	102.63
XS-2022	2.00±0.22	2.02 (3)	159.86 (131.23-192.48)	219.29
ZJ-2017	2.25±0.23	1.79 (3)	5.84 (4.93-6.98)	8.01
ZJ-2018	2.36±0.24	1.75 (3)	14.88 (12.49-17.54)	20.41
ZJ-2019	2.52±0.24	3.41 (3)	18.68 (13.98-24.77)	25.62
ZJ-2020	1.88±0.21	3.15 (3)	34.70 (24.13-48.43)	47.60
ZJ-2021	2.03±0.21	2.34 (3)	91.92 (76.47-111.32)	126.09
ZJ-2022	2.15±0.22	3.40 (3)	101.51 (73.82-140.61)	139.25
JH-2017	2.43±0.24	4.09 (3)	8.79 (6.28-12.05)	12.06
JH-2018	2.12±0.180	9.38 (3)	68.51 (44.33-118.09)	93.98
JH-2019	2.25±0.23	0.77 (3)	71.49 (59.99-84.82)	98.07
JH-2020	2.49±0.24	1.07 (3)	86.89 (73.80-101.84)	119.19
JH-2021	1.96±0.30	0.36 (3)	97.89 (73.96-129.00)	134.28
JH-2022	1.84±0.21	0.26 (3)	133.41 (109.36-165.78)	183.00
LY-2017	2.14±0.22	0.15 (3)	12.75 (10.69-15.38)	17.49
LY-2018	2.14±0.24	2.87 (3)	55.52 (44.92-66.63)	76.16
LY-2019	2.21±0.22	2.99 (3)	59.04 (49.69-70.72)	80.99
LY-2020	2.27±0.24	1.38 (3)	63.95 (52.99-75.86)	87.72
LY-2021	2.46±0.24	1.36 (3)	121.16 (103.24-143.05)	166.20
LY-2022	2.02±0.22	1.11 (3)	259.38 (215.69-316.16)	355.80
NH-2018	2.70±0.26	1.04 (3)	51.39 (44.17-59.80)	70.49
NH-2019	2.41±0.24	2.90 (3)	76.24 (64.70-89.85)	104.58
NH-2020	2.70±0.26	1.26 (3)	91.99 (78.95-106.99)	126.19
WL-2017	1.26±0.19	3.72 (3)	7.54 (4.55-15.98)	10.34
WL-2018	2.47±0.24	1.92 (3)	37.79 (31.95-44.33)	51.84
WL-2019	2.65±0.25	1.60 (3)	76.67 (65.70-89.31)	105.17
WL-2020	2.24±0.23	2.13 (3)	78.91 (65.88-93.63)	108.24

WL-2021	1.87±0.30	0.85 (3)	76.05 (55.59-100.49)	104.32
WL-2022	1.95±0.21	0.71 (3)	106.88 (88.25-129.90)	146.61
YQ-2017	1.63±0.20	2.22 (3)	80.14 (63.23-99.74)	109.93
YQ-2018	2.27±0.23	2.60 (3)	93.10 (78.29-110.36)	127.71
YQ-2019	2.14±0.22	0.40 (3)	99.02 (82.77-118.35)	135.83
YQ-2020	2.23±0.23	0.56 (3)	94.91 (79.72-112.80)	130.19
YQ-2021	2.03±0.23	0.56 (3)	130.94 (124.86-138.97)	179.62
YQ-2022	2.07±0.23	3.35 (3)	167.90 (122.40-251.86)	230.32
RA-2018	1.71±0.20	0.82 (3)	98.71 (79.56-122.31)	135.40
RA-2019	2.01±0.21	0.71 (3)	89.21 (73.63-107.41)	122.37
RA-2020	2.10±0.22	0.88 (3)	76.41 (63.04-91.42)	104.81
RA-2021	2.54±0.35	0.04 (3)	80.25 (63.40-100.39)	110.08
RA-2022	2.25±0.23	3.61 (3)	110.63 (80.83-154.33)	151.76

a Chi-square testing linearity of dose-mortality responses.

b Resistance ratio. The susceptibility baseline to methoxyfenozide (0.729 mg/L) was from reference of Lu et al. (2017).

Table S4 ESTs enzyme activities of *C. suppressalis* in eight field populations.

	Enzyme activity (nmol/min/mg protein)							
	XS	ZJ	JH	LY	NH	WL	YQ	RA
2017	45.24±5.05aA	51.87±2.96aA	50.42±2.81aA	49.31±2.64aA		51.00±3.97aA	52.39±5.18aA	
2018	48.37±3.01aA	46.06±6.87aA	58.84±2.64aA	43.00±3.56aA	47.28±3.57aA	51.23±3.20aA	45.50±2.98aA	47.92±1.35aA
2019	44.57±3.36aA	40.21±4.33aA	59.02±3.04aA	53.37±3.71aA	39.20±2.35aA	52.67±5.40aA	50.02±4.03aA	58.86±4.16aA
2020	55.20±4.32aAB	43.12±4.50aB	49.07±2.26aAB	50.12±1.44aAB	56.06±3.46aAB	52.40±2.55aAB	55.91±1.49aA	56.05±6.24aA
2021	45.03±3.79aA	47.27±4.60aA	45.00±5.03aA	53.43±4.64aA		46.55±6.10aA	48.52±5.84aA	48.15±5.97aA
2022	49.07±1.51aA	45.06±5.59aA	48.43±4.85aA	57.36±5.88aA		57.74±5.87aA	44.22±2.84aA	51.12±2.04aA
Region				*				
Year				ns				
Year × Region				ns				

Standard errors were based on three replicates; Means followed by the same letter within a column do not differ by Tukey's test (*P < 0.05, **P < 0.01).

Capital letters represent differences among different regions in the same year; lowercase letters represent differences among different years in the same region.

Table S5 GSTs enzyme activities of *C. suppressalis* in eight field populations.

	Enzyme activity (nmol/min/mg protein)							
	XS	ZJ	JH	LY	NH	WL	YQ	RA
2017	64.69±8.41aA	60.60±7.72aA	74.61±4.93aA	67.01±4.80aA		63.74±4.66aA	60.56±7.37aA	
2018	66.21±8.00aA	67.24±5.43aA	69.08±6.77aA	73.35±9.80aA	68.08±4.49aA	66.00±4.57aA	65.07±4.95aA	68.89±5.72aA
2019	80.45±4.59aA	74.55±4.37aA	67.41±7.59aA	67.27±6.70aA	66.86±4.91aA	69.94±3.24aA	75.58±3.82aA	68.11±5.58aA
2020	76.58±6.03aA	57.00±4.86aA	69.38±6.80aA	68.63±3.82aA	69.29±7.40aA	72.14±6.21aA	81.51±5.02aA	63.77±4.72aA
2021	63.37±5.90aA	66.63±7.96aA	63.13±6.09aA	75.57±5.24aA		76.03±5.03aA	60.72±2.91aA	69.85±4.70aA
2022	80.43±8.04aA	76.59±10.32aA	72.12±7.76aA	71.85±9.38aA		69.86±4.54aA	77.51±7.07aA	61.35±3.19aA
Region	ns							
Year	ns							
Year × Region	ns							

Standard errors were based on three replicates; Means followed by the same letter within a column do not differ by Tukey's test (*P < 0.05, **P < 0.01).

Capital letters represent differences among different regions in the same year; lowercase letters represent differences among different years in the same region.

Table S6 P450s enzyme activities of *C. suppressalis* in eight field populations.

	Enzyme activity (nmol/min/mg protein)							
	XS	ZJ	JH	LY	NH	WL	YQ	RA
2017	27.01±1.79aAB	18.48±1.13bB	33.10±3.47aA	33.64±2.50abA		29.96±3.12aAB	32.18±3.20aA	
2018	21.27±3.05aA	30.62±1.80aA	34.38±3.33aA	30.07±3.41abA	21.62±1.66bA	33.40±2.64aA	27.16±3.17aA	23.86±3.24bA
2019	25.74±1.62aAB	23.79±1.36abB	34.45±1.85aA	19.79±2.00bB	21.83±2.22bB	29.71±3.90aAB	24.02±1.33aAB	23.30±1.93bB
2020	24.04±2.86aBC	22.00±2.34abC	28.72±3.93aABC	36.38±1.95aAB	39.31±3.06aA	21.80±2.33aC	30.56±3.19aABC	28.53±1.70abABC
2021	22.26±1.53aAB	23.00±2.00abAB	23.89±2.32aAB	28.59±3.76abAB		31.88±2.98aAB	20.45±2.51aB	33.51±1.71aA
2022	20.07±2.03aB	21.57±2.66abAB	25.88±3.22aAB	34.65±1.94abA		24.34±1.98aAB	29.25±4.86aAB	25.17±0.45abAB
Region	**							
Year	*							
Year × Region	**							

Standard errors were based on three replicates; Means followed by the same letter within a column do not differ by Tukey's test (*P < 0.05, **P < 0.01).

Capital letters represent differences among different regions in the same year; lowercase letters represent differences among different years in the same region.

Table S7 Sampling sites, collection dates and developmental stages of *C. suppressalis* collected from fields.

Population	Location	Map ref. No.	Collection date	Stage
XS-2017	Xiaoshan, Zhejiang	①	Jul 3, 2017	adults
XS-2018	Xiaoshan, Zhejiang	①	Apr 4, 2018	adults
XS-2019	Xiaoshan, Zhejiang	①	Aug 3, 2019	adults
XS-2020	Xiaoshan, Zhejiang	①	Mar 29, 2020	adults
XS-2021	Xiaoshan, Zhejiang	①	Jul 9, 2021	adults
XS-2022	Xiaoshan, Zhejiang	①	Aug 11, 2022	pupae
ZJ-2017	Zhuji, Zhejiang	②	Jul 5, 2017	adults
ZJ-2018	Zhuji, Zhejiang	②	Aug 10, 2018	adults
ZJ-2019	Zhuji, Zhejiang	②	Aug 7, 2019	adults
ZJ-2020	Zhuji, Zhejiang	②	Apr 13, 2020	adults
ZJ-2021	Zhuji, Zhejiang	②	July 10, 2021	adults
ZJ-2022	Zhuji, Zhejiang	②	Aug 19, 2022	adults
JH-2017	Jinhua, Zhejiang	③	Jul 21, 2017	adults
JH-2018	Jinhua, Zhejiang	③	Apr 14, 2018	adults
JH-2019	Jinhua, Zhejiang	③	Jul 8, 2019	adults
JH-2020	Jinhua, Zhejiang	③	Apr 20, 2020	adults
JH-2021	Jinhua, Zhejiang	③	Mar 25, 2021	larvae
JH-2022	Jinhua, Zhejiang	③	Jul 8, 2022	adults
LY-2017	Longyou, Zhejiang	④	Jul 28, 2017	adults
LY-2018	Longyou, Zhejiang	④	Aug 2, 2018	adults
LY-2019	Longyou, Zhejiang	④	Jul 11, 2019	adults
LY-2020	Longyou, Zhejiang	④	Mar 26, 2020	larvae
LY-2021	Longyou, Zhejiang	④	Mar 14, 2021	larvae
LY-2022	Longyou, Zhejiang	④	Mar 21, 2022	larvae
NH-2018	Ninghai, Zhejiang	⑤	Mar 28, 2018	adults
NH-2019	Ninghai, Zhejiang	⑤	Apr 2, 2019	adults
NH-2020	Ninghai, Zhejiang	⑤	Apr 5, 2020	adults
WL-2017	Wenling, Zhejiang	⑥	Jul 10, 2017	adults
WL-2018	Wenling, Zhejiang	⑥	Apr 7, 2018	adults
WL-2019	Wenling, Zhejiang	⑥	Aug 15, 2019	adults

WL-2020	Wenling, Zhejiang	⑥	July 13, 2020	adults
WL-2021	Wenling, Zhejiang	⑥	Jul 5, 2021	adults
WL-2022	Wenling, Zhejiang	⑥	Aug 4, 2022	adults
YQ-2017	Yueqing, Zhejiang	⑦	Apr 6, 2017	adults
YQ-2018	Yueqing, Zhejiang	⑦	Mar 30, 2018	adults
YQ-2019	Yueqing, Zhejiang	⑦	Apr 8, 2019	adults
YQ-2020	Yueqing, Zhejiang	⑦	Apr 5, 2020	adults
YQ-2021	Yueqing, Zhejiang	⑦	Apr 10, 2021	adults
YQ-2022	Yueqing, Zhejiang	⑦	Jun 20, 2022	eggs
RA-2018	Ruian, Zhejiang	⑧	Apr 12, 2018	adults
RA-2019	Ruian, Zhejiang	⑧	Apr 6, 2019	adults
RA-2020	Ruian, Zhejiang	⑧	Mar 28, 2020	adults
RA-2021	Ruian, Zhejiang	⑧	Mar 25, 2021	adults
RA-2022	Ruian, Zhejiang	⑧	Mar 22, 2022	adults
